# Knowledge, practice, and attitudes regarding breast cancer self-examination among women of reproductive age in Saudi Arabia: a community-based study

**DOI:** 10.25122/jml-2024-0357

**Published:** 2024-12

**Authors:** Mohammad Ahmad Alenezi, Nasir Ahmed Ali, Abdalaziz Samran Alanzi, Zaid Mohammad Alqahtani, Ashwaq Aiyad Alshammari, Refah Alsubaie, Meshari Sulaiman Bin Huwaymil, Aloush Alotaibi, Ashwaq Mohammad Alrashidi, Muna Mutlaq Alshammari, Tahreer Mutlaq Alshammari, Abdulaziz Ibrahim ltammami

**Affiliations:** 1 Public Health Department, Maternity and Children Hospital-Arar, Arar, Saudi Arabia; 2 Public Health Department, College of Nursing and Health Sciences, Jazan University, Jazan, Saudi Arabia; 3 Executive Management for Community Health, 3rd Health Cluster, Ministry of Health, Riyadh, Saudi Arabia; 4 Iraq Primary Care Center, 3rd Health Cluster, Ministry of Health, Riyadh, Saudi Arabia; 5 Primary Healthcare, Hail Health Cluster, Ministry of Health, Hail, Saudi Arabia; 6 College of Medicine, Majmaah University, Al Majma'ah, Saudi Arabia

**Keywords:** knowledge, practice, attitude, breast cancer self-examination, women of reproductive age, Saudi Arabia

## Abstract

Breast cancer is the second most common cancer among females worldwide and can often be detected at an early stage through breast self-examination (BSE). However, in many developing countries, most cases are diagnosed at advanced stages. This study aimed to assess the knowledge and practice of BSE among women of reproductive age in Saudi Arabia. This study adopted a community-based descriptive-analytical cross-sectional design. A stratified simple random sampling technique was used, with 50 participants from each region. Almost 500 Saudi women who fulfilled the inclusion criteria were enrolled in this study, and of these, 32.8% were within the age group of 20–29, 30.4% were within the age group of 30–39, and 32.8% were within the age group of more than 40 years. The overall knowledge score regarding BSE items among participants was 38%. Significant associations were found between breast cancer knowledge scores and demographic factors such as age group, educational level, marital status, region, and residence area (*P* < 0.05). Regarding knowledge of breast cancer risk factors, 48% of participants demonstrated poor knowledge, 43% had moderate knowledge, and only 9% had good knowledge. Effective prevention of breast cancer requires awareness and understanding of its risk factors. It is important for young women, starting from puberty, to be educated about potential changes in breast tissue and to receive proper training in BSE techniques from healthcare professionals.

## INTRODUCTION

Breast cancer (BC) is the most common malignancy among women worldwide, with over 2.1 million new cases reported in 2018, accounting for approximately one in four cancer diagnoses in women [1,2]. To reduce the mortality rate of cancer, early detection is essential [3,4]. Evidence indicates that since 2008, the mortality rate from cancer has increased by about 14%, with developing countries experiencing higher rates because of delayed diagnosis and inadequate treatment [5]. BC screening methods include mammography, clinical breast exams, and breast self-examinations (BSE) [6]. Despite the availability of international screening guidelines, their implementation remains suboptimal [7]. The World Health Organization (WHO) suggests against using BSE as a screening test for breast cancer. However, it can increase knowledge about what constitutes a normal breast.

The World Bank has categorized Saudi Arabia as having a high-income economy and the 10th lowest poverty rate in the world [8]. Saudi Arabia, classified as a high-income economy by the World Bank, has an age-standardized incidence rate of 27.3 and a mortality rate of 7.5 per 100,000 women for BC, making it the leading cancer among women in the country [9]. Recent studies indicate an increasing trend in BC prevalence among Saudi women [10-12].

Furthermore, cultural and societal barriers, such as the preference for female healthcare providers, misconceptions about BC as divine retribution, and stigma surrounding cancer, often delay diagnosis and hinder treatment [13-15]. Therefore, quick action is needed to raise awareness and support early detection. Numerous preventative measures and early detection techniques are advised to aid in lowering the incidence of certain cancer types [16-20]. The early detection of malignancy, hyperplasia, or aberrant tissue increases the likelihood of therapy success [21-23]. Screening methods, including mammograms and BSE, can identify cancer before symptoms appear, allowing timely intervention [24]. Unfortunately, many patients are diagnosed at advanced stages of the disease when symptoms become apparent, reducing the efficacy of treatments and the likelihood of survival [23,25,26].

Screening tests, therefore, serve as proactive measures in asymptomatic populations, identifying abnormalities before they progress to critical stages [26]. Numerous studies have demonstrated that early screening programs improve survival rates and are more cost-effective than late-stage treatments [27,25]. Cancer screening methods should be affordable and non-invasive, reducing mortality by detecting the disease early [20]. For individuals at high risk of lung cancer, screening with low-dose computed tomography (CT) is recommended, primarily based on age and smoking history [23]. Similarly, asymptomatic women can undergo screening mammograms, which use X-rays to detect cancer at stages too small to be felt by either the patient or a physician [18]. Early detection of small breast cancers through mammography has been shown to significantly reduce cancer-related deaths and morbidity [5]. For colorectal cancer, a variety of screening tests are available, including stool tests, colonoscopy, CT colonography, and flexible sigmoidoscopy [19,25,27]. The success of any cancer screening program, including for breast cancer, relies heavily on public knowledge and awareness [25]. Understanding and addressing gaps in community knowledge about cancer screening can help health authorities improve screening initiatives and campaigns, ultimately leading to higher participation rates and earlier detection.

This study aimed to understand the gaps in knowledge and practices surrounding BSE among Saudi women of reproductive age. It aimed to provide valuable insights into the factors influencing breast cancer awareness, which can inform public health interventions and educational campaigns aimed at improving early detection practices and ultimately reducing breast cancer mortality. This study is important because it addresses the critical issue of late-stage breast cancer diagnoses in developing countries, where early detection through methods like BSE can significantly improve survival rates. By assessing the knowledge and practice of BSE among women of reproductive age in Saudi Arabia, the study highlights gaps in awareness and provides valuable insights into how educational interventions can be designed to empower women with the skills needed to detect potential breast cancer early. Given that breast cancer is the second most common cancer among women globally, improving BSE practices can lead to earlier diagnoses, better treatment outcomes, and reduced mortality, making this research crucial for enhancing public health strategies in Saudi Arabia and similar regions. This study aimed to assess the knowledge, perception, and practice of BSE and BC risk factors among women of reproductive age in Saudi Arabia.

## MATERIAL AND METHODS

### Study design and population

This community-based, descriptive, and analytical cross-sectional study targeted Saudi women of reproductive age (15–49 years) and included participants from all regions of Saudi Arabia. A convenience sampling technique was used to select participants who were readily accessible and willing to participate in the research. Several strategies were employed to minimize participant bias in this study. Random sampling, specifically stratified random sampling, ensured a representative sample from different age groups, educational backgrounds, and regions, reducing selection bias. The sample size was calculated using the following equation:

$$\text{n}=\frac{z^{2}pq}{m^{2}}$$

where z = 1.96 (for 95% confidence level), p = 60% (according to a previous study [28]), q = 40 % (complement of p), and m = 0.05 (margin of error), resulting in a required sample size of 369.

To increase the validity and convenience of the analysis, 500 women were selected. Participants were included if they were Saudi nationals aged 15–49 and willing to participate.

### Research instrument

Data were collected using an online questionnaire distributed via Google Forms through social media platforms. The questionnaire was divided into two sections: the first focused on demographic characteristics and health history, and the second assessed participants’ knowledge and intentions regarding BSE. A pilot study involving 30 participants was conducted to test the reliability of the questionnaire. The questionnaire yielded a Cronbach’s alpha value of 0.978, indicating excellent internal consistency. Data from the pilot study were excluded from the main analysis.

### Data analysis

After data collection, the data were analyzed using SPSS version 24. Qualitative data were summarized as frequencies and percentages and means and standard deviations for quantitative data. One-way ANOVA was used to examine the statistical associations between participants' demographic characteristics and BC knowledge scores. It was also used to assess the relationship between previous BC diagnoses, regular BSE practices, public practices regarding BC screening in Saudi Arabia, and participants' BC knowledge scores. A significance level of *P* ≤ 0.05 was set, with the null hypothesis rejected if this threshold was met.

## RESULTS

As shown in Table 1, most participants were within the age group of 20–29 years (32.8%), 30–39 years (30.4 %), and over 40 years (32.8%), with only 4% under the age of 20 years. About two-thirds of the participants (64.0%) held a bachelor’s degree, and nearly one-quarter were postgraduates (23.6%). More than half (54.4%) were married, and 36.0% were single. About 42.8% of participants were employees, 23.6% were students, 20.0% were employees, and only 13.6% were unemployed. Due to stratified random sampling, we selected an equal number of participants (20%) from each region. Only (15.6%) were from rural areas, and 84.4% from urban areas.

Statistical analysis revealed significant associations between participants’ knowledge scores and demographic variables, including age group, education level, marital status, region, and residence area (*P* < 0.05). However, no significant association was found between occupation and knowledge scores (*P* > 0.05).

**Table 1:** Knowledge scores according to demographic characteristics (*n* = 500)

Demographic characteristics				One way ANOVA			
Variable	Value	*n*	%	Knowledge score		F	*P*
				Mean	SD		
Age group in years	< 20	20	4.0	0.20	0.41	4.19	0.006
	20 - 29	164	32.8	0.34	0.48		
	30 - 39	152	30.4	0.17	0.38		
	> 40	164	32.8	0.26	0.44		
Education level	Secondary or below	62	12.4	0.13	0.34	3.59	.028
	Bachelor’s degree	320	64.0	0.29	0.45		
	Postgraduate	118	23.6	0.24	0.43		
Marital status	Single	180	36.0	0.27	0.44	2.90	0.035
	Married	272	54.4	0.28	0.45		
	Divorced	32	6.4	0.06	0.25		
	Widowed	16	3.2	0.13	0.34		
Occupation	Household	100	20.0	0.28	0.28	0.88	0.450
	Student	118	23.6	0.22	0.22		
	Employee	214	42.8	0.28	0.28		
	Unemployment	68	13.6	0.21	0.21		
Region	Northern Region	100	20.0	0.24	0.43	3.68	0.006
	Middle Region	100	20.0	0.28	0.45		
	Eastern Region	100	20.0	0.12	0.33		
	Western Region	100	20.0	0.32	0.47		
	Southern Region	100	20.0	0.32	0.47		
Residence area	Rural	78	15.6	0.46	0.5	21.28	0.000
	Urban	422	84.4	0.22	0.41		

Table 2 shows that 2.8% of participants had a prior diagnosis of breast cancer, and 17.6% reported a family history of BC. However, only 23.6% of participants performed regular breast self-examinations. There was a statistically significant association between having a prior BC diagnosis, regularly performing breast self-examinations, and BC knowledge scores (*P* < 0.05). However, no significant association was found between a family history of BC and knowledge scores (*P* > 0.05).

**Table 2:** Knowledge scores according to personal and family history (*n* = 500)

Personal and family history				One way ANOVA			
Variable	Value	*n*	%	Knowledge score		F	*P*
				Mean	SD		
Previously diagnosed with BC	Yes	14	2.8	0.46	0.5	28.792	.000
	No	486	97.2	0.22	0.41		
Family history of BC	Yes	88	17.6	0.25	0.46	.871	.351
	No	412	82.4	0.3	0.43		
Regular breast self-exams	Yes	118	23.6	0.36	0.48	8.199	.004
	No	382	76.4	0.23	0.42		

As shown in Figure 2, the most frequently mentioned factors for reducing breast cancer risk were regular breast examination, a healthy diet, and decreased exposure to environmental pollutants.

**Figure 1:**
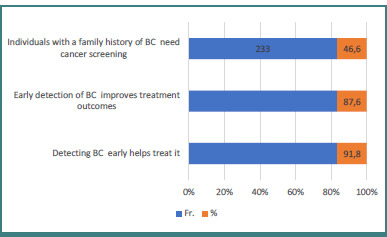
Benefits of BC screening

**Figure 2:**
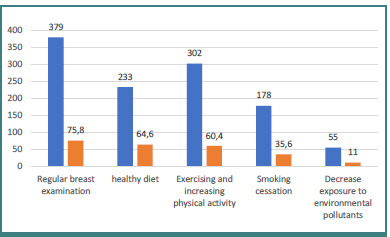
Factors that reduce the risk of BC

**Figure 3:**
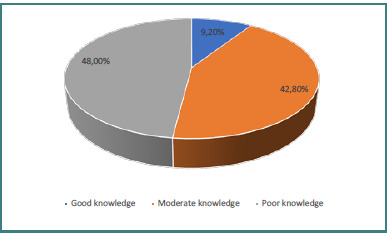
Participants’ assessment related to knowledge of the breast cancer risks

As shown in Figure 3, knowledge scores were categorized into poor, moderate, and good: 48% of participants demonstrated poor knowledge, 43% moderate knowledge, and only 9% good knowledge.

Only 50% of participants correctly mentioned that most breast lumps are found by the women themselves, 17% correctly mentioned that postmenopausal women should perform BSE once a month, about 58% of participants correctly mentioned that abnormal breast change include lumps, hard knots, and the thickening and dimpling of the skin. In comparison, only 25% of participants correctly mentioned that a suitable age for a woman to begin BSE was 20. Participants' overall knowledge regarding BSE items was 38% (Table 3).

**Table 3:** Participants’ knowledge of breast self-examination items

Most breast lumps are found by	Fr.	%
Women themselves (correct)	252	50.4
Physician	84	16.8
Mammography	110	22.0
I don’t know	54	10.8
If the woman is postmenopausal, how often should she do a breast self-examination?		
Once every one month (Correct)	86	17.2
Once every three months	126	25.2
I don’t know	288	57.6
Abnormal breast change		
Lump, hard knot, or thickening	86	17.2
Dimpling of skin	126	25.2
All of the above (Correct)	288	57.6
At what age should a woman begin breast self-examination?		
20 (Correct)	126	25.2
30	2	.4
Don’t know	372	74.4
The overall knowledge		38%

**Figure 4:**
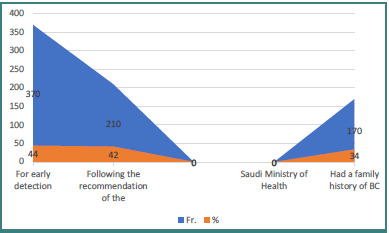
Reasons for undergoing BC screening

As shown in Figure 4, the primary reasons for undergoing BC screening among participants were early detection (44%), following the recommendation of the Saudi Ministry of Health (42%), and having a family history of BC (34%).

Only 26% of participants reported having undergone BC screening in the past, and 22% had conducted BC screening within the last 12 months (Table 4). The main reasons mentioned for not undergoing BC screening were the absence of symptoms (46%), being too young (23%), and a lack of time (16%). Also, there was a statistically significant association between the time since the last screening, the reasons for not undergoing BC screening, and the knowledge scores of BC (*P* < 0.05). However, there was no statistically significant association between those previously diagnosed with BC and knowledge scores (*P* > 0.05).

**Table 4:** Breast cancer screening practices and their association with knowledge scores

Demographic characteristics			One-way ANOVA test			
Practice	No	%	Knowledge score		F	*P*
			Mean	SD		
Previously experienced BC screening						
No	370	74.0	0.24	0.43	1.214	.271
Yes	130	26.0	0.29	0.46		
Time since the last screening?						
<1 year	112	22.4	0.41	0.49	8.061	.000
1–4 years	82	16.4	0.20	0.4		
5–10 years	46	9.2	0.35	0.48		
>10 years	260	52.0	0.19	0.39		
Reasons for not undergoing BC screening						
No symptoms	228	45.6	0.36	0.48	13.267	.000
Still young	114	22.8	0.04	0.18		
Lack of time	80	16.0	0.33	0.47		
Fear of screening results	32	6.4	0.31	0.47		
Don’t know screening settings	46	9.2	0.13	0.34		

As shown in Figure 1, participants highlighted several benefits of BC screening. The majority (91.8%) stated that early detection of cancer aids in its treatment, while 87.6% mentioned that early detection improves treatment outcomes. Additionally, 46.6% recognized the importance of cancer screening for individuals with a family history of BC.

## DISCUSSION

This community-based descriptive-analytical cross-sectional study was used to assess the knowledge, perception, and practice of BSE and BC risk factors among women of reproductive age in Saudi Arabia. Almost 500 Saudi females who fulfilled the inclusion criteria were enrolled in this study. Of these, 32.8% were within the age group of 20-29, 30.4% were within the age group of 30–39, and 32.8% were within the age group of more than 40 years. Two-thirds of participants (64.0%) held a bachelor’s degree, and nearly one-quarter were postgraduates (23.6%). More than half of the participants (54.4%) were married, and 36.0% were single. About 42.8% of the participants were employees, 23.6% were students, 20.0% were employees, and only 13.6% were unemployed. Using stratified random sampling, an equal number of participants (20%) were selected from each region. In addition, when comparing the participants’ knowledge with their demographic characteristics, there was a statistically significant association between age group, educational level, marital status, region, residential area, and knowledge scores (*P* < 0.05). However, no significant association was observed between occupation and knowledge scores (*P* > 0.05). The overall knowledge score of the participants regarding BSE items was 38%. Only 50% of participants correctly stated that most breast lumps are found by women themselves, and just 17% correctly identified that postmenopausal women should perform BSE once every month. Approximately 58% of participants recognized abnormal breast changes, including lumps, hard knots, and skin thickening or dimpling. Furthermore, only 25% correctly identified 20 years as the appropriate age for women to begin BSE. These findings align with previous studies [28]. For example, another study by Tomic *et al*. reported that 94% of women demonstrated good knowledge about BSE [29]. Awareness of risk factors is essential for the prevention of breast cancer. Regarding factors that reduce the risk of BC, three-quarters of participants listed routine breast exams, maintaining a healthy diet and reducing exposure to environmental toxins.

In terms of knowledge about BC risks, participants in this study were categorized as having poor (48%), moderate (43%), or good knowledge (9%). While early BC screening has proven benefits, it is not without group harms, such as overdiagnosis and false positives. These disparities in the benefits and risks have led to variations in major screening guidelines. Furthermore, individual risk levels (average vs. high risk) and screening methods influence evidence-based recommendations [27]. Tomic *et al*. found that female medical students had satisfactory knowledge of BC [29]. Another study emphasized that the evidence on overdiagnosis does not imply that breast screening lacks value. Instead, the trade-off between the benefits and potential harms of breast cancer screening, such as false positives and overdiagnosis, is more nuanced and balanced than previously understood [26]. It is essential for young women to understand the potential for changes in breast tissue and to receive professional training in BSE methods, starting at puberty. While most breast alterations are benign, women should consult a doctor if they notice any changes in either breast. Initially, a diagnostic mammography may be recommended [28]. Regarding the benefits of breast cancer screening, participants in this study highlighted several key advantages. Most (91.8%) agreed that early detection helps treat cancer, followed by 87.6% who noted that early detection improves treatment outcomes. Additionally, 46.6% mentioned that individuals with a family history of BC should undergo screening. Perceived susceptibility scored the lowest mean among participants, while perceived benefits had the highest mean. A statistically significant correlation (*P* < 0.01) was observed between BSE intention and self-efficacy, as well as perceived barriers and advantages.

Improving awareness and addressing factors influencing women's behavior regarding BC screening is vital for effective prevention. Researchers can design and implement successful behavioral change interventions by focusing on these factors [30]. Interventions based on theory-centered determinants are expected to yield better outcomes [26]. One study conducted in Iran found that women who participated in BSE had much better self-efficacy and perceived benefits than those who did not. Fewer perceived impediments were noted by those who performed BSE [31]. Additionally, the results showed that only 26% of participants had previously undergone breast cancer (BC) screening, with 22% reporting screening within the last 12 months. The primary reasons cited for not undergoing BC screening were the absence of symptoms (46%), feeling too young (23%), and lack of time (16%). These findings are consistent with a 2024 study, which reported that only 35% of surveyed women regularly practiced breast self-examinations [32]. There was a statistically significant association between the time since the last screening, reasons for not undergoing breast cancer screening, and knowledge scores (*P* < 0.05). However, no significant association was found between previous BC diagnosis and knowledge scores (*P* > 0.05), aligning with findings from previous studies [28,31]. Finally, this study extends the existing literature by providing a comprehensive assessment of BSE knowledge and practices among women of reproductive age in Saudi Arabia, a region where breast cancer is a leading cause of mortality. While much of the literature focuses on the general knowledge and practices of BSE, this study specifically examines the influence of socio-demographic factors such as age, education, and region on BSE awareness and practices. It also adds new insights into the relationship between previous breast cancer diagnoses, regular engagement in self-examination, and public attitudes toward breast cancer screening in Saudi Arabia. By highlighting these associations, the study underscores the need for tailored educational interventions to improve early detection and prevention efforts. Additionally, it provides valuable context for understanding how cultural and regional factors shape health behaviors, which can inform future public health strategies aimed at reducing late-stage breast cancer diagnoses in developing countries.

### Limitations

Our study is not without limitations. First, the study’s reliance on cross-sectional data may have limited the interpretation of any causal association. Lastly, the sample design and varying response rates among the selected colleges may limit the generalizability of this research.

## CONCLUSION

The study found a lack of public knowledge about BC screening, especially regarding methods to reduce BC risk. Participants with a family history of BC showed a much higher awareness and knowledge. In addition, the study also identified inadequate BC screening behaviors among participants, highlighting the need for immediate intervention. Preventing BC requires a clear understanding of its risk factors, and it is essential to educate young women, starting from puberty, about recognizing changes in breast tissue. Training by healthcare professionals on the proper BSE techniques can further improve early detection practices. This study adds to the limited research on preventive breast cancer practices in Saudi Arabia. Educational efforts should focus on enhancing women's understanding of self-screening methods while also improving access to information about the availability of free screenings and mammograms. Furthermore, a well-publicized screening program with an easy registration process is crucial for increasing participation.

## Data Availability

The data supporting this study are available from the corresponding author upon reasonable request.

## Personal thanks

The authors sincerely thank all participants for their cooperation and valuable contributions to this study.
